# Rapid, High-Throughput Identification of Anthrax-Causing and Emetic Bacillus cereus Group Genome Assemblies via BTyper, a Computational Tool for Virulence-Based Classification of Bacillus cereus Group Isolates by Using Nucleotide Sequencing Data

**DOI:** 10.1128/AEM.01096-17

**Published:** 2017-08-17

**Authors:** Laura M. Carroll, Jasna Kovac, Rachel A. Miller, Martin Wiedmann

**Affiliations:** Department of Food Science, Cornell University, Ithaca, New York, USA; Rutgers, The State University of New Jersey

**Keywords:** Bacillus cereus group, taxonomy, virulence genes, whole-genome sequencing

## Abstract

The Bacillus cereus group comprises nine species, several of which are pathogenic. Differentiating between isolates that may cause disease and those that do not is a matter of public health and economic importance, but it can be particularly challenging due to the high genomic similarity within the group. To this end, we have developed BTyper, a computational tool that employs a combination of (i) virulence gene-based typing, (ii) multilocus sequence typing (MLST), (iii) *panC* clade typing, and (iv) *rpoB* allelic typing to rapidly classify B. cereus group isolates using nucleotide sequencing data. BTyper was applied to a set of 662 B. cereus group genome assemblies to (i) identify anthrax-associated genes in non-B. anthracis members of the B. cereus group, and (ii) identify assemblies from B. cereus group strains with emetic potential. With BTyper, the anthrax toxin genes *cya*, *lef*, and *pagA* were detected in 8 genomes classified by the NCBI as B. cereus that clustered into two distinct groups using *k*-medoids clustering, while either the B. anthracis poly-γ-d-glutamate capsule biosynthesis genes *capABCDE* or the hyaluronic acid capsule *hasA* gene was detected in an additional 16 assemblies classified as either B. cereus or Bacillus thuringiensis isolated from clinical, environmental, and food sources. The emetic toxin genes *cesABCD* were detected in 24 assemblies belonging to *panC* clades III and VI that had been isolated from food, clinical, and environmental settings. The command line version of BTyper is available at https://github.com/lmc297/BTyper. In addition, BMiner, a companion application for analyzing multiple BTyper output files in aggregate, can be found at https://github.com/lmc297/BMiner.

**IMPORTANCE** Bacillus cereus is a foodborne pathogen that is estimated to cause tens of thousands of illnesses each year in the United States alone. Even with molecular methods, it can be difficult to distinguish nonpathogenic B. cereus group isolates from their pathogenic counterparts, including the human pathogen Bacillus anthracis, which is responsible for anthrax, as well as the insect pathogen B. thuringiensis. By using the variety of typing schemes employed by BTyper, users can rapidly classify, characterize, and assess the virulence potential of any isolate using its nucleotide sequencing data.

## INTRODUCTION

The Bacillus cereus group, also known as Bacillus cereus sensu lato (*s.l*.), consists of nine closely related bacterial species: B. anthracis (1), B. cereus sensu stricto (*s.s*.), B. cytotoxicus (2), B. mycoides (3), B. pseudomycoides (4), B. thuringiensis, B. toyonensis (5), B. weihenstephanensis (3), and B. wiedmannii (6). The pathogenic potentials of members of the B. cereus group vary widely; while some isolates are capable of causing anthrax or anthrax-like disease (7), foodborne illness (8), or food spoilage issues (9–11), others are used in industrial settings as probiotics (5, 12–14), insecticides and pest control agents (15), agents in environmental pollutant bioremediation (15–17), plant growth promoters (15, 18), and even as producers of bacteriocins (19, 20) or parasporins with anticancer activities (15, 21, 22). As the industrial and agricultural applications of these microorganisms expand, differentiating between isolates that can cause anthrax or gastrointestinal illness and those that can be used as beneficial microbes in industrial or agricultural settings becomes critical. Relying strictly on taxonomic classification at the species level can lead not only to isolate misclassification, but also to an inaccurate assessment of a given isolate's virulence potential. There have been numerous cases in which probiotics containing B. cereus group isolates sold for human and/or animal consumption were found to possess strains capable of producing toxins Nhe and/or Hbl (12, 14, 23), or the species they contained were incorrectly identified (12, 14, 24). Additionally, B. thuringiensis, a biopesticide, can possess B. cereus s.s. toxin genes and potentially infect humans via the food chain (25), a notable example being a foodborne outbreak associated with salad that was potentially caused by B. thuringiensis serovar aizawai that had been sprayed on a produce field (26).

Differentiating between pathogenic and nonpathogenic B. cereus group isolates is a matter of public health and economic importance but can be a challenging task. Phenotypic and biochemical methods (27), as well as many commonly used molecular methods, such as 16S rRNA gene sequencing, may not have sufficient discriminatory power to differentiate between members of the B. cereus group (28, 29). In addition, the ability of a particular B. cereus group isolate to cause disease in humans is not species dependent, and taxonomic classification can often be a poor predictor of an isolate's virulence potential (30); for example, genes encoding diarrheal toxins have been found in B. cereus, B. mycoides, B. pseudomycoides, B. thuringiensis, and B. weihenstephanensis (30–32). For these reasons, better tools are needed to classify B. cereus isolates, from both taxonomical and food safety risk perspectives (33).

A number of genetic loci have been proposed as markers that can be used to taxonomically classify and/or differentiate between pathogenic and nonpathogenic B. cereus group isolates at greater resolution than phenotypic methods and 16S rRNA gene sequencing (30). Some examples of taxonomic markers include the housekeeping gene *rpoB* (6, 30, 34–38), the pantoate-beta-alanine ligase gene *panC* (39–43), and multiple loci used in a 7-gene multilocus sequence typing (MLST) scheme (i.e., *glp*, *gmk*, *ilv*, *pta*, *pur*, *pyc*, and *tpi*) (30, 44–49) (https://pubmlst.org/bcereus/). Each of these methods alone provides greater resolution than its predecessors, and the methods may be implemented in combination with each other and/or with phenotypic methods (30, 33, 40, 49).

The presence and absence of virulence and toxin genes have also served as indicators in a method by which B. cereus group isolates can be classified as pathogenic or nonpathogenic (28, 30, 50). These methods are beneficial from a clinical perspective, as genes associated with many medically relevant phenotypes are plasmid carried (51), including anthrax toxin and capsule genes (52), and *ces* genes, which encode cereulide synthetase (53). This can be contrasted with the fact that many genes that encode phenotypic traits used to distinguish members of the B. cereus group using biochemical and microbiological tests are contained on the chromosome (motility, hemolysis, etc.) (51). As a result, a disease phenotype, such as the ability to cause anthrax-like symptoms in a particular host (52), may not be confined to a single B. cereus group species, making species-level taxonomy a poor indicator of an isolate's pathogenic potential.

Molecular typing methods using housekeeping and virulence genes found in members of the B. cereus group have been essential for classifying isolates from both a taxonomical and a public health perspective. However, as whole-genome sequencing (WGS) becomes cheaper, faster, and more accessible, the ability to perform molecular typing methods *in silico* becomes even more attractive. With the goal of creating a readily accessible open-source pipeline that can be easily used by B. cereus researchers and public health officials, we have created BTyper, a computational tool to perform (i) virulence gene detection, (ii) MLST, (iii) *panC* clade typing, and (iv) *rpoB* allelic typing using B. cereus group nucleotide sequencing data in either FASTA, SRA, or gzipped FASTQ format. Additionally, we applied BTyper and BMiner, a companion application for analyzing BTyper's output files in aggregate, to a set of 662 B. cereus group genome assemblies, with the goal of identifying (i) anthrax-associated genes in non-anthracis Bacillus members of the B. cereus group, and (ii) assemblies from B. cereus group strains with emetic potential.

## RESULTS

### Construction and validation of BTyper using *in vitro* methods.

**FIG 1:**
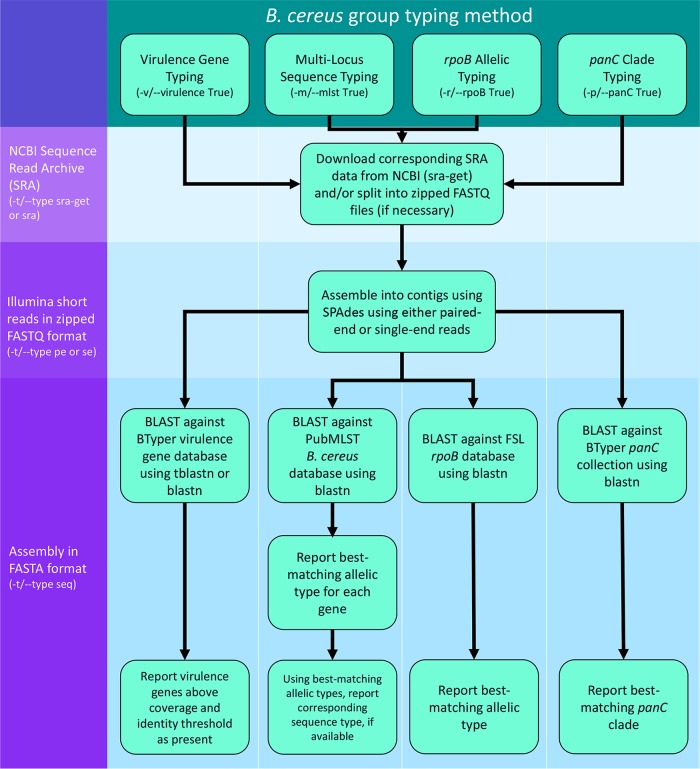
BTyper command line workflow for various types of data and default typing methods. Input datum type is listed in the left margin, while typing methods are listed at the top of the chart. Command line parameters associated with a particular typing method are shown in parentheses. FSL, Food Safety Lab.

BTyper was used to perform *in silico* (i) virulence gene detection, (ii) MLST, (iii) *panC* clade typing, and (iv) *rpoB* allelic typing using the default settings described in Materials and Methods. Both assembled genomes and Illumina paired-end reads from 46 B. cereus group genomes were used (Fig. 1). BTyper was successfully able to predict *rpoB* allelic types and whole-genome phylogenetic clade using *panC* for all B. cereus group genomes tested (*n* = 46; Table 1). For *in silico* MLST, it was successful at predicting the sequence type in all but one isolate (45 out of 46; Table 1); isolate FSL M8-0091 was the only isolate for which *in silico* prediction of sequence type did not match the sequence type obtained by Sanger sequencing. For this isolate, the only allele that differed between the two methods was the *tpi* allele: Sanger sequencing yielded a *tpi* allelic type of 20, while BTyper's *in silico* prediction was *tpi* allelic type 175, which was a perfect match and differed from *tpi* 20 by a single nucleotide at position 284. However, SRST2 (54) also obtained a *tpi* allelic type of 175, making it likely that (i) the colony selected to undergo WGS had a different *tpi* allele than the colony selected to undergo Sanger sequencing, or (ii) there was an error in either WGS or Sanger sequencing.

**TABLE 1:** Percentage of isolates in which BTyper correctly identified the presence/absence of eight virulence genes, MLST, *rpoB* AT, and *panC* clade

Data set	Virulence gene (%)^*a*^								MLST ST (%)^*b*^	*rpoB* AT (%)^*c*^	*panC* clade (%)^*d*^
	*hblA*	*hblC*	*hblD*	*nheA*	*nheB*	*nheC*	*cytK*	*entFM*			
Training (*n* = 22)											
Assemblies	100	100	100	100	95.5	100	90.9	95.5	100	100	100
PE reads^*e*^	100	90.9	100	90.9	95.5	95.5	90.9	95.5	100	100	100
Validation (*n* = 24)											
Assemblies	91.7	100	95.8	87.5	95.8	100	100	91.7	95.8	100	100
PE reads	91.7	100	91.7	87.5	95.8	100	100	91.7	95.8	100	100
Total (*n* = 46)											
Assemblies	95.7	100	97.8	93.5	95.7	100	95.7	93.5	97.8	100	100
PE reads^*e*^	95.7	95.7	95.7	89.1	95.7	97.8	95.7	93.5	97.8	100	100

a Presence/absence of eight virulence genes from previously published WGS data (training set) or PCR (validation set).

b Multilocus sequence typing (MLST) results from previously published WGS data (training set) or Sanger sequencing (validation set).

c *rpoB* allelic typing (AT) results from previously published WGS data (training set) or Sanger sequencing (validation set).

d *panC* clade typing results from previously published WGS data.

e Illumina paired-end (PE) reads.

For virulence gene detection, the results obtained from BTyper matched the PCR results for eight selected virulence genes in over 89% of all isolates (*n* = 46; Table 1). This resulted in an overall sensitivity and specificity of 99.0% and 85.5%, respectively, when the default parameters for assembled genomes were used, and an overall sensitivity and specificity of 97.0% and 85.5%, respectively, when default parameters for Illumina paired-end reads were used.

### Characteristics associated with B. cereus group phylogenetic clade III are most prevalent among genome assemblies currently available at NCBI.

**FIG 2:**
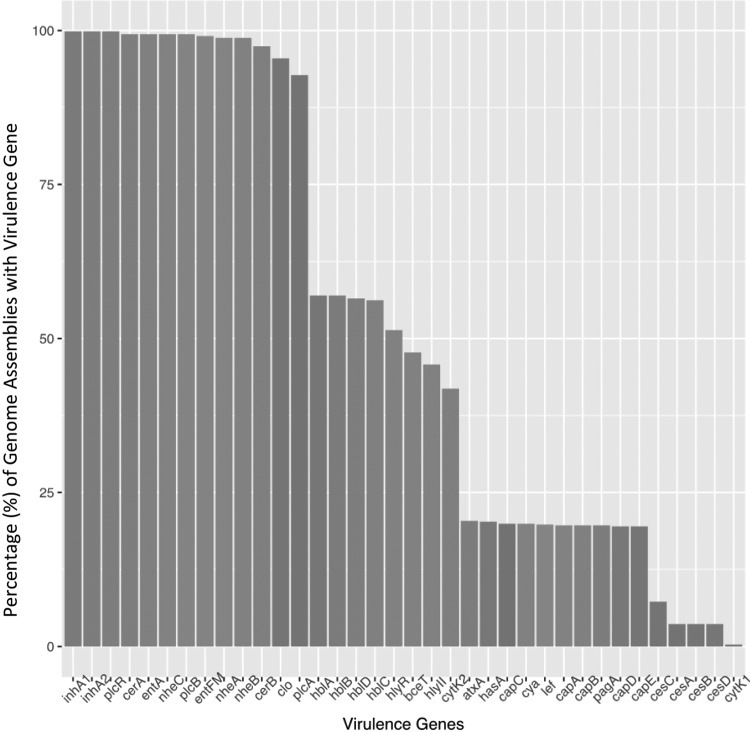
Percentage (%) of B. cereus group assemblies in which a particular virulence gene was detected. Minimum identity and coverage thresholds of 50 and 70%, respectively, were used for virulence gene detection.

BTyper was used to perform virulence gene detection, MLST, *panC* clade typing, and *rpoB* allelic typing on 662 B. cereus group genome assemblies (157 assemblies labeled as B. anthracis, 353 assemblies as B. cereus s.s., 2 assemblies as B. cytotoxicus, 19 assemblies as B. mycoides, 2 assemblies as B. pseudomycoides, 94 assemblies as B. thuringiensis, 3 assemblies as B. toyonensis, 21 assemblies as B. weihenstephanensis, and 11 assemblies as B. wiedmannii). Within the 662 assemblies, 13 virulence genes were detected in more than 90% of all genomes when the default minimum amino acid sequence identity and coverage thresholds of 50 and 70% were used, respectively (Fig. 2). The least commonly detected gene was *cytK1* (Fig. 2), which was detected in both available B. cytotoxicus genomes and no other WGS assemblies.

For *in silico* MLST, 544 assemblies were assigned to one of 213 B. cereus sequence types (STs), the most common of which was ST1 (*n* = 123 isolates). This was unsurprising, considering that ST1 is associated with B. anthracis (55), and B. anthracis makes up a considerable portion (23.7%) of the B. cereus group genome assemblies currently in NCBI's database. *In silico rpoB* allelic typing grouped the 662 isolates into one of 43 different, best-matching *rpoB* allelic types (ATs), with 185 isolates matching AT463 most closely. AT463 has been previously associated with clade III isolates (30), the phylogenetic clade that encompasses B. anthracis.

**FIG 3:**
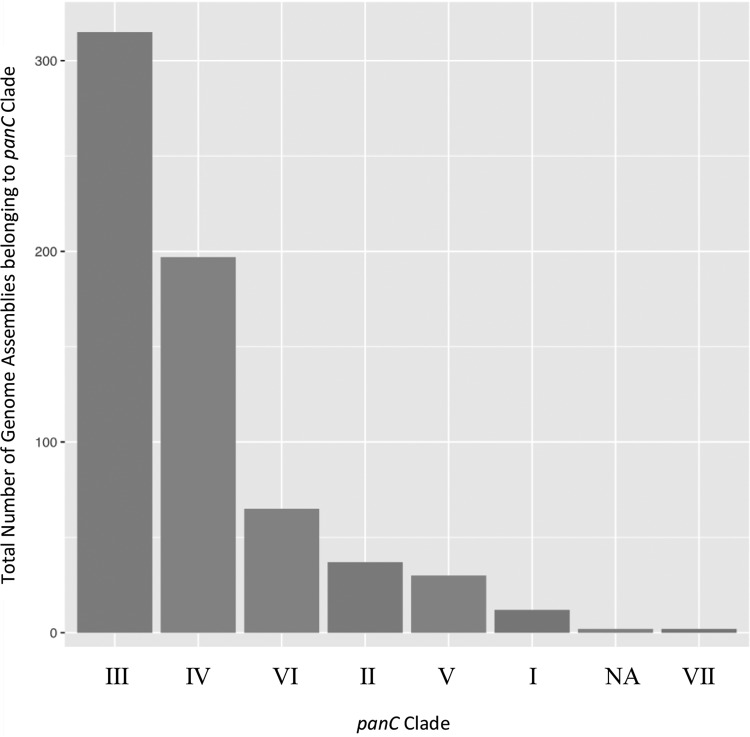
Closest-matching phylogenetic clade using the *panC* loci from 662 B. cereus group genome assemblies. A *panC* locus could not be assigned in 4 genome assemblies, which is denoted by “NA.”

For *panC*-based phylogenetic clade typing, a *panC* locus was detected in 658 out of 662 genomes (Fig. 3). The most commonly assigned clade was clade III, a polyphyletic clade which contains B. anthracis, as well as some strains currently misclassified in the NCBI database as B. cereus s.s. and B. thuringiensis (30, 39, 40). Together, clade IV, which consists of some B. cereus s.s. and B. thuringiensis strains (30, 39, 40), as well as the type strains of these two species, and clade III accounted for more than 75% of all B. cereus group WGS assemblies in the NCBI database (Fig. 3). Clade VII, which contains the B. cytotoxicus (2) type strain, was the most poorly represented clade; the two available B. cytotoxicus assemblies were placed here.

### Application of BTyper to identify B. anthracis-associated genes in non-anthracis Bacillus isolates reveals virulence gene heterogeneity within genome assemblies from anthrax toxin-encoding isolates.

When Fisher's exact test was used to determine if any virulence genes were significantly associated with a phylogenetic clade, virulence genes typically associated with B. anthracis were found to be significantly associated with members of clade III after a Bonferroni correction was applied (*P* < 0.05; Table 2). The B. anthracis toxin genes *cya* (edema factor-encoding), *lef* (lethal factor-encoding), and *pagA* (protective antigen-encoding), as well as their regulator gene *atxA* (56), were found only in clade III isolates (*P* < 0.05; Table 2). In addition, B. anthracis polyglutamate capsule synthesis genes *capABCDE* (57) were more commonly associated with clade III assemblies (*P* < 0.05; Table 2) and found primarily in genomes classified in the NCBI database as B. anthracis. Meanwhile, genes associated with diarrheal disease (8) were found to be significantly associated with clades II, IV, V, and VI (*P* < 0.05; Table 2); these included the diarrheal toxin genes *hblCDAB*, which were found to be significantly associated with clades II, IV, V, and VI (*P* < 0.05; Table 2), while being less common in members of clade III (*P* < 0.05; Table 2), driven by the large number of B. anthracis assemblies in this clade that did not possess these genes.

**TABLE 2:** Virulence genes significantly associated with 5 B. cereus group phylogenetic clades after a Bonferroni correction^*a*^

Clade	Genes
II	*hblCDAB*
III	*atxA*,^*b*^ *capABCDE*, *cya*,^*b*^ *hasA*, *hlyII*, *hlyR*, *lef*,^*b*^ *pagA*^*b*^
IV	*bceT*, *cytK2*, *hblCDAB*
V	*bceT*, *hblCDAB*^*c*^
VI	*bceT*, *cesC*, *hblCDAB*^*c*^

a Significant at a *P* value of <0.05. For exact corrected *P* values, see Table S7.

b Indicates a virulence gene that was detected only in its respective clade (includes clades I and VII).

c Indicates a virulence gene that was detected in all members of its respective clade.

**FIG 4:**
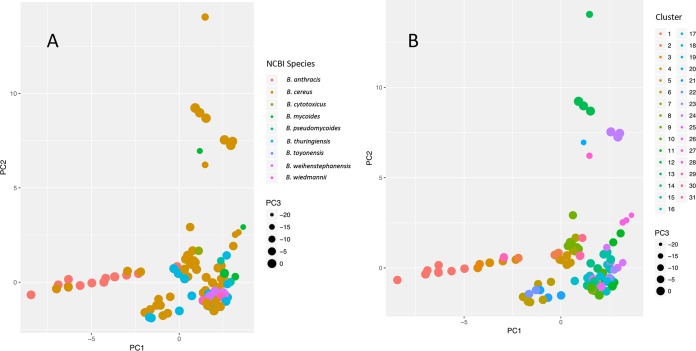
Principal-component analysis (PCA) of 662 B. cereus group genome assemblies based on presence/absence of virulence genes. Virulence gene typing was carried out using BTyper, while PCA was performed using BMiner. Principal components 1 (PC1) and 2 (PC2) are plotted on the *x* and *y* axes, respectively, while principal component 3 (PC3) corresponds to point size. Plots are colored by isolate species, as found in NCBI (A), and assigned cluster using *k*-medoids (B). To view interactive versions of these plots containing isolate names and metadata, all BTyper final results files and metadata can be downloaded from https://github.com/lmc297/BTyper/tree/master/sample_data and viewed in BMiner.

**FIG 5:**
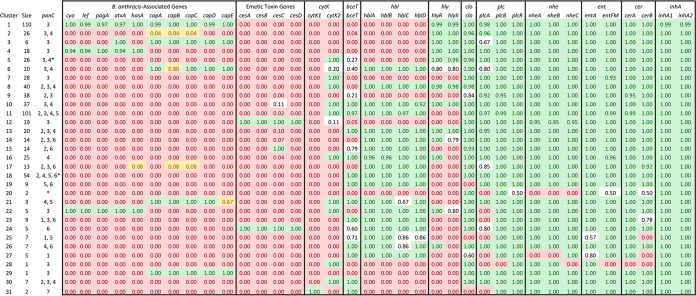
*k*-medoids clusters based on presence/absence of virulence genes detected using BTyper. Size corresponds to the number of assemblies assigned to a given cluster, while *panC* corresponds to *panC* clades found in the cluster, with an asterisk denoting one or more assemblies that could not be placed into a *panC* clade. Numbers within cells correspond to the proportion of assemblies in a given cluster in which the corresponding virulence gene was detected. Green shading corresponds to a virulence gene detected in more than 90% of all assemblies in a cluster, while red shading corresponds to a virulence gene detected in fewer than 10% of all assemblies in a cluster. Yellow shading corresponds to B. anthracis-associated genes detected in fewer than 90% but greater than 0% of assemblies in a cluster.

Principal-component analysis (PCA) based on the presence/absence of virulence genes using BMiner revealed several assemblies labeled as B. cereus and B. thuringiensis that clustered with B. anthracis assemblies (Fig. 4A). When *k*-medoids clustering was performed with an optimum *k* of 31, isolates classified in the NCBI database as B. anthracis were placed into clusters 1 through 8 (Fig. 4B). Additionally, clusters 17, 21, 22, and 29 did not contain any assemblies labeled in NCBI as B. anthracis, but they contained at least one assembly in which one or more of the B. anthracis-associated virulence genes identified using Fisher's exact test were detected (Fig. 5).

**FIG 6:**
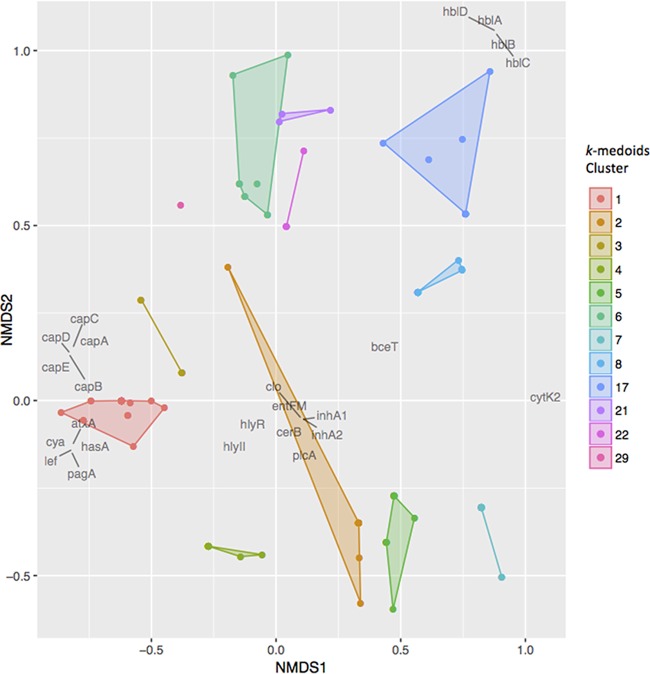
Nonmetric multidimensional scaling (NMDS) plot of Bacillus cereus group clusters that (i) possessed at least one assembly that was classified as Bacillus anthracis in NCBI, and/or (ii) possessed at least one assembly in which at least one B. anthracis-associated virulence gene (*cya*, *lef*, *pagA*, *atxA*, *hasA*, and/or *capABCDE*) was detected using BTyper. NMDS was performed in BMiner using virulence gene presence/absence data and a Jaccard dissimilarity metric. Isolates are represented by points, and convex hulls and shading correspond to the assigned *k*-medoids cluster. Virulence genes are plotted in dark gray.

Cluster 1 (Fig. 4B), which contained the majority of isolates labeled as B. anthracis, contained 110 isolates, 107 of which were classified in the NCBI database as B. anthracis, and all of which belonged to *panC* clade III (Fig. 5). Assemblies derived from human and veterinary clinical isolates associated with anthrax disease populated a large proportion of the cluster, including assemblies associated with isolates from the 2001 anthrax bioterrorism attacks (58), European heroin users and an associated outbreak (59, 60), and a 2011 outbreak in Swedish cattle (61). Three assemblies labeled as B. cereus clustered among them (Fig. 4B). Two of these assemblies were labeled as B. cereus strain 03BB102, an isolate that was thought to cause fatal pneumonia in a welder in San Antonio, TX (Table 3), while the third was labeled as B. cereus biovar anthracis strain CI, which caused fatal anthrax in a chimpanzee in the rainforest of Taï National Park, Côte d'Ivoire (Table 3) (51). Consistent with these findings, placement into cluster 1 was driven largely by an assembly's possession of all, or nearly all, anthrax-associated genes identified using Fisher's exact test (Fig. 6); the anthrax toxin genes *cya*, *lef*, and *pagA*, toxin regulator gene *atxA*, hyaluronic acid capsule gene *hasA*, and B. anthracis polyglutamate capsule genes *capABCDE* were detected in nearly all (>97%) cluster 1 assemblies (Fig. 5).

**TABLE 3:** Non-anthracis Bacillus assemblies in which anthrax toxin genes *cya*, *lef*, and/or *pagA* were detected using BTyper

Cluster^*a*^	NCBI species classification	*panC* clade^*b*^	GenBank accession no.^*c*^	Strain	Isolate source (reference)	Gene(s) detected?					
						*cya*	*lef*	*pagA*	*atxA*	*hasA*	*capABCDE*
1	B. cereus	III	GCA_000022505.1, GCA_000832405.1	03BB102	Human with fatal pneumonia, San Antonio, TX, USA (104)	+	+	+	−	+	+
1	B. cereus	III	GCA_000143605.1	Biovar anthracis strain CI	Chimpanzee with fatal anthrax, Taï National Park, Côte d'Ivoire (51)	+	+	+	+	+	+
22	B. cereus	III	GCA_000167215.1, GCA_000832805.1	G9241	Human with pneumonia, nausea, and vomiting, LA, USA (65)	+	+	+	+	+	−
22	B. cereus	III	GCA_000688755.1	BcFL2013	Human with anthrax-like skin lesion, FL, USA (66)	+	+	+	+	+	−
22	B. cereus	III	GCA_000789315.1	03BB87	Human with fatal pneumonia, Lubbock, TX, USA (67)	+	+	+	+	+	−
22	B. cereus	III	GCA_002007005.1	LA2007	Human with fatal pneumonia and septic shock, Galliano, LA, USA (68)	+	+	+	+	+	−

a Clusters were assigned using a *k*-medoids approach (*k* = 31).

b *panC* clades were assigned using BTyper.

c Multiple accession numbers are given for strains associated with multiple assemblies.

Despite the fact that all assemblies classified in NCBI as B. anthracis were assigned to clusters 1 through 8, the only other clusters in addition to cluster 1 in which anthrax toxin genes were detected were clusters 4 and 22. Like cluster 1, all isolates in clusters 4 and 22 belonged to *panC* clade III, and nearly all possessed the anthrax toxin genes *cya*, *lef*, and *pagA*, regulator gene *atxA*, and hyaluronic acid capsule gene *hasA* (Fig. 5). However, the B. anthracis polyglutamate capsule genes *capABCDE* were not detected in any of the cluster 4 or cluster 22 assemblies at the default identity and coverage thresholds (Fig. 5). While cluster 4 (*n* = 18; Fig. 4B) contained only isolates classified in the NCBI database as B. anthracis, it contained assemblies from several strains with attenuated virulence, including several vaccine strains (62–64). Cluster 22 (*n* = 5; Fig. 4B), however, contained 5 anthrax-associated assemblies, all of which were classified in the NCBI database as B. cereus (Table 3). All assemblies in cluster 22 originated from human clinical isolates in which the isolate was classified as B. cereus, but the patient presented anthrax-like symptoms; two assemblies were of B. cereus strain G9241, a strain of Bacillus isolated from the sputum and blood of a patient with pneumonia, nausea, and vomiting (65). The isolate, which had been classified as B. cereus via biochemical tests and 16S rRNA gene sequencing, was found to possess the anthrax toxin gene *pagA* but not the polyglutamate capsule genes *capABCDE* (65), which is consistent with its classification using BTyper (Table 3). BTyper's classification of the three other assemblies in this cluster also aligned with their previously published descriptions and included the following: (i) a B. cereus assembly associated with an isolate from a patient in Florida possessing an anthrax-like skin lesion (66), which was found to possess anthrax toxin genes *cya*, *lef*, and *pagA* and the hyaluronic acid capsule gene *hasA* and belong to ST78 (66), (ii) a B. cereus isolate from a patient with a fatal case of pneumonia in Lubbock, TX, that was also found to possess B. anthracis virulence genes (67), and (iii) an assembly associated with a B. cereus isolate that was found to possess anthrax toxin genes and *hasA* and was isolated from a patient in Galliano, LA, who had a fatal case of pneumonia and septic shock (Table 3) (68).

While no anthrax toxin genes were detected outside clusters 1, 4, and 22, other B. anthracis-associated genes identified using Fisher's exact test were detected in several other clusters and assemblies. Cluster 3 (*n* = 6; Fig. 4B) contained 6 B. anthracis assemblies belonging to *panC* clade III in which the B. anthracis toxin regulator gene *atxA* and polyglutamate capsule genes *capABCDE* were detected (Fig. 5). Other assemblies in this cluster included B. anthracis strain Smith 1013, described as “Pasteur-like” in that it possessed plasmid pXO2 (the plasmid associated with *cap* genes) but not plasmid pXO1 (the plasmid associated with B. anthracis toxin genes) (69, 70), as well as B. anthracis strain Pasteur itself (Table 4).

**TABLE 4:** Non-anthracis Bacillus assemblies in which B. anthracis-associated genes were detected, excluding anthrax toxin genes *cya*, *lef*, and *pagA* and regulator *atxA*

Cluster	NCBI species classification	*panC* clade	GenBank accession no.^*c*^	Strain/isolate ID^*a*^	Isolate source (reference)	Gene detected?					
						*hasA*	*capA*	*capB*	*capC*	*capD*	*capE*
2	B. cereus	III	GCA_001286905.1	JRS1	Rhazya stricta rhizosphere, Jeddah, Saudi Arabia (105)	−	+	+	+	−	−
6	B. cereus	III	GCA_000003955.1	AH1273	Human blood, Iceland (52)	−	+	+	+	+	+
6	B. cereus	III	GCA_000161395.1	AH1272	Amniotic fluid, Iceland (52)	−	+	−	+	+	+
6	B. cereus	III	GCA_000181655.1, GCA_000832865.1	03BB108	Dust containing pneumonia-causing B. cereus strain 03BB012 (106)	−	+	+	+	+	+
6	B. cereus	IV	GCA_000398945.1	Schrouff	Food (107)	−	+	+	+	+	+
6	B. cereus	IV	GCA_000399185.1	K-5975c	Food (107)	−	+	+	+	+	+
6	B. cereus	IV	GCA_000399305.1	HuB4-4	Soil, Belgium (107)	−	+	−	+	+	+
6	B. thuringiensis	III	GCA_000161595.1	Serovar Monterrey strain BGSC 4AJ1	Mexico (108)	−	+	+	+	+	+
6	B. thuringiensis	IV	GCA_001640965.1	BGSC 4C1	Bombyx mori, Czechoslovakia (109)	−	+	+	+	+	+
17	B. cereus	VI	GCA_002014585.1	FSL H8-0485	Soil, USA (110)	+	−	−	−	−	−
17	B. thuringiensis	III	GCA_000948155.1	Et10/1	Geothermal spring, Lirima thermal springs, Chile (111)	−	−	+	+	−	−
21	B. cereus	IV	GCA_000161315.1	F65185	Open fracture, NY, USA (112)	−	+	+	+	+	+
21	B. cereus	V	GCA_000290835.1	VD115	Soil, Guadeloupe (107)	−	+	+	+	+	+
21	B. thuringiensis	IV	GCA_001677055.1^*b*^	BGSC 4BT1	Red soil, China (113)	−	+	+	+	+	−
29	B. cereus	III	GCA_001913295.1	MOD1_Bc119	Whole black pepper, USA (114)	−	+	+	+	+	+

a ID, identification.

b *capE* was detected at a lower amino acid identity (47.7%, compared to the default threshold of 50%).

c Multiple accession numbers are given for strains associated with multiple assemblies.

The polyglutamate capsule genes *capABCDE* were also detected in assemblies assigned to clusters 6, 21, and 29 (Table 4). Cluster 6 (*n* = 10; Fig. 4B) contained 10 assemblies: 1 assembly classified in NCBI as B. anthracis, 7 assemblies classified as B. cereus, and 2 assemblies classified as B. thuringiensis. Members of this cluster belonged to *panC* clades III and IV, and consistent with the detection of *cap* genes in this cluster, one of the B. thuringiensis assemblies in this group had been shown to produce a polyglutamate capsule (71). Cluster 21 (*n* = 3; Fig. 4B) contained 2 assemblies labeled as B. cereus and 1 assembly labeled as B. thuringiensis. One of the B. cereus assemblies came from B. cereus strain F65185, which was confirmed to belong to ST168 and was isolated from a patient in New York with an open fracture wound (Table 4). Members of this group belonged to either *panC* clade IV or V. Cluster 29 (*n* = 1; Fig. 4B) consisted of a single B. cereus assembly belonging to *panC* clade III and associated with a strain isolated from whole black pepper in the United States in 2015 (Table 4).

Additionally, *cap* genes were detected in a single isolate in clusters 2 and 17 (*n* = 26 and 13, respectively; Fig. 4B). However, B. anthracis-associated genes were not detected in any other assemblies in this cluster, despite being composed primarily of assemblies classified as B. anthracis (21, 4, and 1 assemblies labeled in NCBI as B. anthracis, B. cereus, and B. thuringiensis, respectively). Consistent with a lack of virulence genes, this cluster contained the genome of the avirulent strain B. anthracis Ames, which is commonly used in laboratory settings and does not possess B. anthracis plasmid pXO1 or pXO2 (72). All non-anthracis Bacillus assemblies in this group were isolated from either food or environmental sources, and all belonged to either *panC* clade III or IV.

### Application of BTyper to identify assemblies associated with emetic B. cereus group isolates.

Assemblies possessing emetic toxin genes *cesABCD* were grouped into two clusters using *k*-medoids. Cluster 12 (*n* = 19; Fig. 4B) consisted of 19 assemblies classified as B. cereus in NCBI. All belonged to *panC* clade III, *cesABCD* were detected in all assemblies, and *hblCDAB* were not detected in any assemblies (Fig. 5). Included in this cluster was strain AH187, an isolate from the United Kingdom that was responsible for a 1972 emetic outbreak (Table 5). This isolate tested positive for emetic toxin (cereulide) formation and nonhemolytic enterotoxin (NHE) and negative for HBL hemolytic enterotoxin and cytotoxin K, and it belonged to MLST ST26 (Table 5) (73); these findings were confirmed using BTyper. Other notable strains in this cluster included (i) emetic strain B. cereus H3081.97, a B. cereus strain of sequence type 144 (ST144) which is closely related to strain AH187, and (ii) emetic strain B. cereus NC7401 (74).

**TABLE 5:** B. cereus group assemblies in which emetic toxin genes *cesABCD* were detected

Cluster	NCBI species classification	*panC* clade	GenBank accession no.	Strain	Isolate source (reference)
12	B. cereus	III	GCA_000021225.1	AH187	Vomit of a person who ate cooked rice; isolate was associated with an emetic outbreak in 1972 (73)
12	B. cereus	III	GCA_000161075.1	BDRD-ST26	BDRD stock strain (52)^*a*^
12	B. cereus	III	GCA_000171035.2	H3081.97	Food; emetic toxin-producing isolate from 1997 outbreak linked to rice, TX, USA (115)
12	B. cereus	III	GCA_000283675.1	NC7401	Emetic isolate (74)
12	B. cereus	III	GCA_000290935.2	IS075	Wild mammal (vole) (116)
12	B. cereus	III	GCA_000290995.1	AND1407	Black currant (53)
12	B. cereus	III	GCA_000291235.1	MSX-A12	Not available (107)
12	B. cereus	III	GCA_000399205.1	IS845/00	Bank vole, Poland (107, 117)
12	B. cereus	III	GCA_000399225.1	IS195	Bank vole, Poland (107, 117)
12	B. cereus	III	GCA_000743195.1	F1-15	Foodborne source (118)
12	B. cereus	III	GCA_001566375.1	MB.15	Food, Munich, Germany (119)
12	B. cereus	III	GCA_001566385.1	MB.18	Food, Munich, Germany (119)
12	B. cereus	III	GCA_001566435.1	MB.16	Food, Munich, Germany (119)
12	B. cereus	III	GCA_001566445.1	MB.17	Food, Munich, Germany (119)
12	B. cereus	III	GCA_001566455.1	MB.21	Food, Munich, Germany (119)
12	B. cereus	III	GCA_001566465.1	MB.8	Food, Munich, Germany (119)
12	B. cereus	III	GCA_001566515.1	MB.8-1	Food, Munich, Germany (119)
12	B. cereus	III	GCA_001566525.1	MB.20	Food, Munich, Germany (119)
12	B. cereus	III	GCA_001566535.1	MB.22	Food, Munich, Germany (119)
24	B. cereus	VI	GCA_000291155.1	MC67	Sandy loam, Møn, Denmark (75, 107, 120)
24	B. cereus	VI	GCA_000291315.1	CER074	Raw milk (53)
24	B. cereus	VI	GCA_000291335.1	CER057	Parsley (53)
24	B. cereus	VI	GCA_000293605.1	BtB2-4	Forest soil (53)
24	B. cereus	VI	GCA_000399245.1	MC118	Sandy loam, Møn, Denmark (75, 107, 120)

a BDRD, Biological Defense Research Directorate.

The other cluster in which all *cesABCD* genes were detected in all assemblies was cluster 24 (*n* = 5; Fig. 4B). This cluster contained 5 assemblies classified as B. cereus, all of which belonged to *panC* clade VI (Table 5). Unlike cluster 12, *hblCDAB* genes were detected in all assemblies in this cluster (Fig. 5). The assemblies in this cluster originated from food and environmental isolates (Table 5). Despite their assemblies being classified in the NCBI database as B. cereus, all 5 strains in this cluster were classified as emetic B. weihenstephanensis in their respective manuscripts, and all were capable of growth at 8°C (53, 75).

## DISCUSSION

### Accessible whole-genome sequence analysis tools can facilitate improved taxonomic classification and characterization of B. cereus group isolate virulence potential.

As whole-genome sequencing becomes more widely used in the realms of public health and food safety, the ability to classify potential pathogenic microorganisms quickly and effectively becomes increasingly important. A number of bioinformatics tools already exist for this purpose, including SRST2, which can be used to perform MLST and detect antimicrobial resistance genes using Illumina reads (54); SeqSero, which performs *in silico* serotyping using Illumina reads or nucleotide assemblies from Salmonella enterica isolates (76); PlasmidFinder, which can be used to detect plasmids in isolates using Illumina reads or nucleotide assemblies (77); and VirulenceFinder, which can be used to detect virulence genes in Listeria monocytogenes, Staphylococcus aureus, Escherichia coli, and Enterococcus (78). Recently, methods such as *in silico* MLST and virulence gene detection have been combined into single computational pipelines that can be used to characterize numerous bacterial species (79). Here, we have created a bioinformatics tool specific to the Bacillus cereus group that combines virulence gene detection using a curated database of B. cereus virulence factors with *in silico* manifestations of established molecular and virulence typing methods to phylogenetically classify and rapidly assess the virulence potential of any B. cereus group isolate. Additionally, we have provided a companion application, BMiner, that allows users to interact with data from hundreds of genomes at once, which we anticipate will become increasingly valuable as more B. cereus group genomes are sequenced.

The *in silico* typing methods employed by BTyper and other bioinformatics tools are valuable from a public health and food safety perspective, due to their (i) speed, as BTyper and similar tools can be used to perform gene detection and typing tasks in seconds using assembled genomes (76, 77); (ii) scalability, with the ability to provide users with information about a single isolate or hundreds from the command line (54, 76); and (iii) ability to output concise and easily interpretable summaries of large amounts of data (54), making it easy for a user to understand their results, share data with colleagues, and make informed decisions about an isolate in question (i.e., is it pathogenic or not). Additionally, the use of virulence gene-based typing as employed by BTyper offers the advantage that isolates can be classified according to their virulence potential, which means that one does not have to make any prior assumptions about the taxonomic classification of an isolate in question. This marks a valuable step forward in distinguishing pathogenic B. cereus group isolates from their nonpathogenic counterparts; however, marked improvements could be made to BTyper and similar tools through the integration of phenotypic data. By associating genotypic characteristics of B. cereus group isolates with phenotypic data, such as host illness and symptoms and growth temperature, BTyper and other tools used to genotype foodborne pathogens may become more valuable from a risk assessment perspective.

### Analysis of publicly available B. cereus group assemblies using BTyper and BMiner identifies virulence gene-based clusters that capture phylogenetic heterogeneity in isolates with similar phenotypes.

Using the output of BTyper and BMiner, virulence gene profiles of 662 B. cereus group genomes were assigned to one of 31 clusters by employing a *k*-medoids approach, without making unnecessary prior assumptions about an assembly's taxonomic classification in the public domain. This allowed for the identification of several well-defined clusters with clinical or taxonomic relevance, including (i) fully virulent B. anthracis and B. anthracis-like B. cereus (cluster 1), (ii) *capABCDE*-negative anthrax-causing B. cereus strains (cluster 22), (iii) B. anthracis with attenuated virulence (clusters 3 and 4), (iv) 2 emetic clusters (clusters 12 and 24), and (v) B. cytotoxicus (cluster 31). The clustering of the emetic assemblies into 2 separate clusters reflected the observed heterogeneity among emetic strains of B. cereus and B. weihenstephanensis: Hoton et al. (53) described two distinct clusters formed by emetic toxin-producing B. cereus group strains, with psychrotolerant B. weihenstephanensis strains belonging to a distinct emetic cluster (referred to in its respective manuscript as cluster II) (53, 80). Assemblies from these strains were placed into a single cluster (*k*-medoids cluster 24) consisting of B. weihenstephanensis assemblies belonging to *panC* clade VI, while members of Hoton et al.'s emetic cluster I were placed into a second cluster (*k*-medoids cluster 12) containing assemblies belonging to *panC* clade III. For B. cytotoxicus, the two available assemblies, both of which were the only *panC* clade VII representatives, were placed into a single cluster composed of only themselves (*k*-medoids cluster 31), driven largely by their possession of *cytK1*, as described by Guinebretière et al. (40). For B. anthracis, strains possessing both anthrax virulence plasmids (pXO1 and pXO2) were assigned to cluster 1, distinguishing them from attenuated strains in which one or neither plasmid was detected, as well as B. cereus strains that caused anthrax-like disease (cluster 22). Despite lacking the polyglutamate capsule genes *capABCDE*, B. cereus strains in cluster 22 were able to cause anthrax-like symptoms using a second capsule encoded by B. cereus exopolysaccharide genes *bpsXABCDEFGH* (*bpsX-H*) on a different plasmid, pBC218 (81). The *bpsX-H* operon in its entirety was detected in 4 of the 5 anthrax-causing, *capABCDE*-negative B. cereus assemblies in cluster 22 (all but strain BcFL2013) and in no other cluster. It is likely that results like this from additional studies will be able to further resolve clade assignments and disease phenotypes with BTyper; recently, Bazinet identified numerous genes associated with phenotypic traits, such as anthrax and food poisoning (82). Here, we found associations between B. cereus group virulence genes and the *panC* clade, and virulence gene heterogeneity within disease phenotypes was identified. As more B. cereus group WGS and associated metadata become available, the potential for identifying new virulence alleles or phylogenetic markers that can further identify alleles or genes that are not only associated with a particular disease, but with specific symptoms or a clinical outcome using BTyper, becomes promising. For example, future work will be needed to better define specific genetic markers that can classify B. cereus group strains and clusters that are likely to cause diarrheal illnesses. Future epidemiological studies that assess the associations between different clusters and disease outcomes and symptoms will also provide an opportunity to further define and refine the types of disease outcomes and public health risks associated with different B. cereus group strains.

## MATERIALS AND METHODS

### Database construction.

To construct a virulence gene database specific to B. cereus group isolates, amino acid sequences from a total of 36 virulence genes (see Table S1 in the supplemental material) were collected from the National Center for Biotechnology Information (NCBI) (https://www.ncbi.nlm.nih.gov/). For an MLST database, the 7-gene MLST database for Bacillus cereus was downloaded from PubMLST (https://pubmlst.org/bcereus/). For *panC* typing, chromosomes of 45 B. cereus group strains were downloaded from the NCBI database (Table S2). *panC* genes were extracted from each strain using nucleotide BLAST (BLASTn) (83) and the *panC* genes of various B. cereus group type strains, and the online tool available at https://tools.symprevius.org/Bcereus/english.php was used to ensure that at least one representative from each of the seven *panC* clades was present in the collection (40) (Table S2). For *rpoB* allelic typing, the *rpoB* allelic type database created and curated by Cornell University's Food Safety Lab and Milk Quality Improvement Program (CUFSL/MQIP; Ithaca, NY) was used. While 16S rRNA gene typing is not performed by default (see “Construction of BTyper tool,” below), 16S rRNA gene typing can be performed using reference 16S rRNA gene sequences from nine different B. cereus group type strain genomes. To obtain these sequences, the 16S rRNA gene sequence from a cultured B. cereus type strain was downloaded from the Ribosomal Database Project (RDP) (84) and used in conjunction with BLASTn (83) to extract 16S rRNA gene genes from each of nine different B. cereus group species type strain genomes (Table S3). All database files can be downloaded from https://github.com/lmc297/BTyper.

### Construction of BTyper tool.

BTyper was created with the following dependencies: Python version 2.7 (https://www.python.org/), Biopython version 1.6.8 (85), BLAST version 2.4.0 (83), SPAdes version 3.9.0 (86), and SRA toolkit version 2.8.0 (87, 88). The whole-genome sequences of 22 previously characterized B. cereus group isolates (30) were downloaded from the NCBI and used as a training set to optimize parameters (referred to here as the “training set”; Table S4). For virulence gene detection using translated nucleotide BLAST (tBLASTn) (83), default minimum coverage and minimum identity thresholds of 70 and 50%, were chosen, respectively, as they correlated highly with previously published PCR results (30), and the allele with the highest corresponding bit score was reported. For MLST, *rpoB* allelic typing, and *panC* clade typing, the highest-scoring allele in the respective database was selected using its associated BLAST bit score, with no minimum threshold applied (Fig. 1). Virulence gene detection, MLST, *rpoB* allelic typing, and *panC* clade typing methods were chosen to be performed by default, as these methods are valuable for their discriminatory power (30). 16S rRNA gene typing, although not performed by default due to its inability to discriminate between phylogenetic clades and species (34, 89, 90), was added as an option as well, as many users may be interested in this locus. For this method, the highest-scoring 16S rRNA gene of the nine type strain 16S rRNA genes was selected using its BLAST bit score, with no minimum threshold applied.

### PCR detection of virulence genes.

To assess the accuracy of BTyper's *in silico* virulence gene detection, each of the 24 isolates in the validation set was screened for eight virulence genes (*hblA*, *hblC*, *hblD*, *nheA*, *nheB*, *nheC*, *cytK*, and *entFM*) using PCR. Bacterial DNA used as the template in PCRs was extracted by inoculating single colonies into 100 μl of sterile water; lysates were then heated at 95°C for 10 min in a thermocycler. For PCRs, 1 μl of dirty lysate was added to a master mix containing sterile water, 2× GoTaq Green master mix (Promega, Madison, WI), and primers at a concentration of 0.4 μM each (Table S5). The PCRs included an initial denaturation time of 3 min at 94°C, followed by 30 cycles of amplification; each cycle consisted of denaturation at 94°C for 30 s, annealing (see Table S5 for annealing temperatures) for 30 s, and elongation for 1 min at 72°C, with a final extension at 72°C for 7 min. PCR products were electrophoresed in 1% agarose gels, followed by ethidium bromide staining to confirm specific amplification. For isolates that did not yield a PCR amplicon for a given gene, the PCR was repeated at least once in order to confirm the negative PCR result.

### MLST.

Multilocus sequence typing (MLST) was performed for all 24 isolates in the validation set using a 7-housekeeping-gene scheme available through the PubMLST website (https://pubmlst.org/bcereus/). The PCRs consisted of 1 μl of dirty lysate as the DNA template added to a master mix containing sterile water, 2× GoTaq Green master mix (Promega), and primers at a final concentration of 0.4 μM each. The PCR cycles included an initial denaturation (3 min at 94°C), followed by 20 cycles of denaturation (94°C for 30 s), annealing for 30 s with a touchdown scheme (annealing temperatures that decrease by 0.5°C per cycle, starting with 55°C and reaching 45°C at the last cycle), and elongation at 72°C for 45 s. The 20 cycles of touchdown PCR were followed by an additional 20 cycles using an annealing temperature of 45°C. A final extension at 72°C for 5 min was included at the end of the 40 cycles. After amplification, the PCR products were sequenced at the Biotechnology Resource Center (BRC; Cornell University, Ithaca, NY), and ATs and sequence types (STs; based on all 7 genes) were assigned using the PubMLST website. All isolates were submitted to the B. cereus PubMLST database (30).

### *rpoB* allelic typing.

A 632-nucleotide (nt) internal sequence of *rpoB*, encoding the β-subunit of the RNA polymerase, was used for assigning *rpoB* allelic types (ATs), as described previously (11). The sequences of all *rpoB* ATs are available in the Food Microbe Tracker database (91).

### Validation of BTyper using additional B. cereus group whole-genome sequences.

The genomes of 24 additional B. cereus group isolates were sequenced and assembled according to Miller et al. (referred to here as the “validation set”; Table S6) (6). BTyper was used to perform virulence gene detection, MLST, *rpoB* allelic typing, and *panC* clade typing on each draft genome using the chosen default settings (see “Construction of BTyper tool,” above). The same analyses were performed using the Illumina paired-end reads associated with each isolate, again using BTyper's default settings. To assess the accuracy of the *panC* clades assigned by BTyper, clade assignments provided by BTyper were compared to the isolates' whole-genome sequence clades provided by Kovac et al. (30) and Miller et al. (R. A. Miller, J. Jian, S. M. Beno, L. M. Carroll, M. Wiedmann, and J. Kovac, unpublished data) for the training and validation sets, respectively. A current version of the command line tool, as well as the curated virulence gene and *rpoB* allelic type databases, can be found at https://github.com/lmc297/BTyper. A link to a Web-based version of BTyper will also be made available at https://github.com/lmc297/BTyper at a later time.

### Construction of BMiner companion application.

BMiner, a companion application for parsing, viewing, and analyzing multiple BTyper files in aggregate, was created with the following dependencies: R version 3.3.2 (92) and R packages shiny version 1.01 (93), ggplot2 version 2.2.1 (94), readr version 1.1.0 (95), stringr version 1.2.0 (96), vegan version 2.4-2 (97), plyr version 1.8.4 (98), dplyr version 0.5.0 (99), cluster version 2.0.6 (100), ggrepel version 0.6.5 (101), and magrittr version 1.5 (102). BMiner is freely available at https://github.com/lmc297/BMiner.

### Application of BTyper and BMiner to whole-genome sequencing data.

The latest assembly versions for all (*n* = 651) B. cereus group genome assemblies available in GenBank were downloaded on 6 April 2017. Genome assemblies were assigned to one of nine taxa according to their GenBank classification: B. anthracis (*n* = 157), B. cereus s.s. (*n* = 343), B. cytotoxicus (*n* = 2), B. mycoides (*n* = 19), B. pseudomycoides (*n* = 2), B. thuringiensis (*n* = 93), B. toyonensis (*n* = 3), B. weihenstephanensis (*n* = 21), and B. wiedmannii (*n* = 11). BTyper was used to perform virulence gene detection, MLST, *rpoB* allelic typing, and *panC* clade typing on all 651 isolates, as well as an additional 11 isolates that were part of the validation set but did not have assemblies in the NCBI database at the time (total number of B. cereus group genomes, 662). All available metadata associated with each assembly's BioSample were downloaded from the NCBI (103). Data mining using BTyper results from all 662 B. cereus group assemblies was conducted using BMiner. The final results files for all 662 B. cereus group genome assemblies, as well as the associated metadata, can be found at https://github.com/lmc297/BTyper.

### *Post hoc* statistical analyses.

*Post hoc* statistical analyses were conducted in R version 3.3.2 (92). Fisher's exact test was used to test for associations between virulence genes and *panC*-based phylogenetic clades using the fisher.test function in R's stats package (Table S7). Phylogenetic clades I and VII were excluded from this analysis, due to both being underrepresented among B. cereus group genomes in the NCBI database (12 and 2 isolates, respectively), while rare and common virulence genes present in fewer than 20 and more than *n* − 20 assemblies (where *n* corresponds to the total number of assemblies being tested), respectively, were also excluded. A Bonferroni correction was used to correct for multiple comparisons. To find members of the B. cereus group that clustered with B. anthracis isolates based on their virulence gene presence-absence profiles, as well as to assess within-species virulence heterogeneity, *k*-medoids clustering was performed using the clara function in R's cluster package (100) and a Euclidean distance metric. To find an optimum value for *k*, *k*-medoids clustering was performed for each value of *k* for 2 ≤ *k* ≤ (*n* − 1), where *n* is 662, the total number of assembled genomes. A *k* value of 31 was selected, as it corresponded to the largest average silhouette width.

## Supplementary Material

Supplemental material
