# Supplementary material for: Rapid, High-Throughput Identification of Anthrax-Causing and Emetic Bacillus cereus Group Genome Assemblies via BTyper, a Computational Tool for Virulence-Based Classification of Bacillus cereus Group Isolates by Using Nucleotide Sequencing Data
Source: Appl Environ Microbiol. 2017 Aug 17;83(17):e01096-17. doi: 10.1128/AEM.01096-17 (PMC5561296; doi:10.1128/AEM.01096-17)
Supplement: Supplemental material [file supp_83_17_e01096-17__index.html]

Supplemental material 

# Rapid, High-Throughput Identification of Anthrax-Causing and Emetic Bacillus cereus Group Genome Assemblies via BTyper, a Computational Tool for Virulence-Based Classification of Bacillus cereus Group Isolates by Using Nucleotide Sequencing Data

## Supplemental material

- Supplemental file 1 -

  Amino acid sequences (Table S1), *B. cereus* group genomes used for *panC* database construction (Table S2), *B. cereus* group genomes used for 16S rRNA gene database construction (Table S3), *B. cereus* group isolate genomes in the training set (Table S4), primers (Table S5), *B. cereus* group genomes in the validation set (Table S6), and virulence genes (Table S7).

  PDF, 147K
