# Supplementary material for: Rapid, High-Throughput Identification of Anthrax-Causing and Emetic Bacillus cereus Group Genome Assemblies via BTyper, a Computational Tool for Virulence-Based Classification of Bacillus cereus Group Isolates by Using Nucleotide Sequencing Data
Source: Appl Environ Microbiol. 2017 Aug 17;83(17):e01096-17. doi: 10.1128/AEM.01096-17 (PMC5561296; doi:10.1128/AEM.01096-17)
Supplement: Supplemental material [file AEM.01096-17_zam999118007s1.pdf]

Supplemental Table S1. Amino acid sequences of *B. cereus* group virulence genes used for virulence typing.

Amino Acid Sequence	NCBI Accession Number	Description
AtxA	10956365	Anthrax toxin regulator, pXO1-118 (plasmid) [*Bacillus anthracis*]
BceT	1665720	Diarrheal toxin [*Bacillus cereus*]
CapA	10956446	Poly-γ-D-glutamate capsule biosynthesis, pXO2-56 (plasmid) [*Bacillus anthracis*]
CapB	10956448	Poly-γ-D-glutamate capsule biosynthesis, pXO2-58 (plasmid) [*Bacillus anthracis*]
CapC	10956447	Poly-γ-D-glutamate capsule biosynthesis, pXO2-57 (plasmid) [*Bacillus anthracis*]
CapD	753753473	Capsule biosynthesis protein CapD (plasmid) [*Bacillus anthracis*]
CapE	821583427	Polyglutamate capsule biosynthesis protein CapE [*Bacillus anthracis*]
CerA	505581110	Cereolysin A [*Bacillus cereus*]
CerB	505581118	Cereolysin B [*Bacillus cereus*]
CesA	190015727	Cereulide synthetase A, CesA (plasmid) [*Bacillus cereus*]
CesB	190015609	Cereulide synthetase B, CesB (plasmid) [*Bacillus cereus*]
CesC	190015504	ABC transporter ATP-binding protein, CesC (plasmid) [*Bacillus cereus*]
CesD	190015506	Putative permease, CesD (plasmid) [*Bacillus cereus*]
Clo	62550724	Cereolysin O [*Bacillus cereus*]
Cya	10956369	Anthrax toxin edema factor, pXO1-122 (plasmid) [*Bacillus anthracis*]
CytK1	114152686	Cytotoxin K1 [*Bacillus cytotoxicus*]
CytK2	30019265	Cytotoxin K2 [*Bacillus cereus* ATCC 14579]
EntA	30023271	Enterotoxin / cell-wall binding protein [*Bacillus cereus* ATCC 14579]
EntFM	30020092	Enterotoxin [*Bacillus cereus* ATCC 14579]
HasA	10956340	Hyaluronic acid capsule, pXO1-93 (plasmid) *[Bacillus anthracis*]
HblA	30021214	Hemolysin BL binding component precursor [*Bacillus cereus* ATCC 14579]
HblB	30021213	Hemolysin BL binding component precursor [*Bacillus cereus* ATCC 14579]
HblC	30021216	Hemolysin BL lytic component L2 [*Bacillus cereus* ATCC 14579]
HblD	30021215	Hemolysin BL lytic component L1 [*Bacillus cereus* ATCC 14579]
HlyII	30021625	Hemolysin II [*Bacillus cereus* ATCC 14579]
HlyR	31322346	Hemolysin II regulatory protein [*Bacillus cereus*]
InhA1	30019435	Immune inhibitor A precursor [*Bacillus cereus* ATCC 14579]
InhA2	30018848	Immune inhibitor A precursor [*Bacillus cereus* ATCC 14579]
Lef	10956354	Anthrax toxin lethal factor, pXO1-107 (plasmid) [*Bacillus anthracis*]
NheA	30019951	Non-hemolytic enterotoxin lytic component L2 [*Bacillus cereus* ATCC 14579]
NheB	30019952	Non-hemolytic enterotoxin lytic component L1 [*Bacillus cereus* ATCC 14579]
NheC	30019953	Enterotoxin C [*Bacillus cereus* ATCC 14579]
PagA	4894326	Anthrax toxin protective antigen, pXO1-110 (plasmid) [*Bacillus anthracis*]
PlcA	30021854	1-phosphatidylinositol phosphodiesterase precursor [*Bacillus cereus* ATCC 14579]
PlcB	30018852	Phospholipase C [*Bacillus cereus* ATCC 14579]

PlcR	358355901	PlcR [*Bacillus cereus* NC7401]

Supplemental Table S2. *B. cereus* group genomes used for *panC* database construction.

NCBI Accession Number	Isolate	*panC* Clade
NZ_CP007618.1	*B. anthracis* 2000031021	3
NZ_CP009902.1	*B. anthracis* 2002013094	3
NZ_CP001974.1	*B. anthracis* A16R	3
NZ_AAAC02000001.1	*B. anthracis* A2012	3
NC_007530.2	*B. anthracis* Ames Ancestor	3
NC_012472.1	*B. cereus* 03BB102	3
NZ_CP009941.1	*B. cereus* 03BB87	3
NZ_CM000717.1	*B. cereus* 172560W	4
NC_011658.1	*B. cereus* AH187	3
NC_011773.1	*B. cereus* AH820	3
NZ_CM000715.1	*B. cereus* ATCC 10876	4
NC_003909.8	*B. cereus* ATCC 10987	3
NC_004722.1	*B. cereus* ATCC 14579	4
NZ_CM000721.1	*B. cereus* ATCC 4342	3
NC_011725.1	*B. cereus* B4264	4
NC_014335.1	*B. cereus* biovar anthracis CI	3
NC_006274.1	*B. cereus* E33L	3
NZ_CM001787.1	*B. cereus* F	3
NZ_CM000736.1	*B. cereus* F65185	4
NZ_CP008712.1	*B. cereus* FT9	3
NZ_CM000714.1	*B. cereus* m1293	3
NC_011969.1	*B. cereus* Q1	3
NZ_ACMH00000000.1	*B. cereus* Rock1-15	4
NZ_CM000732.1	*B. cereus* Rock3-42	3
NZ_CM000734.1	*B. cereus* Rock4-2	4
NC_009674.1	*B. cytotoxicus* NVH 391-98	7
NZ_CM000745.1	*B. pseudomycoides* DSM 12442	1
NC_008600.1	*B. thuringiensis* Al Hakam	3
NZ_CM000754.1	*B. thuringiensis* serovar andalousiensis BGSC 4AW1	3
NC_017200.1	*B. thuringiensis* serovar finitimus YBT-020	3
NZ_CP004870.1	*B. thuringiensis* serovar galleriae HD-1	4
NZ_CM000756.1	*B. thuringiensis* serovar huazhongensis BGSC 4BD1	4
NZ_CP004858.1	*B. thuringiensis* serovar konkukian YBT-1520	4
NC_005957.1	*B. thuringiensis* serovar kurstaki 97-27	3
NZ_CP010089.1	*B. thuringiensis* serovar kurstaki HD-29	4
NZ_CM000751.1	*B. thuringiensis* serovar kurstaki T03a001	4
NZ_CM000752.1	*B. thuringiensis* serovar monterrey BGSC 4AJ1	3

NZ_CM000750.1	*B. thuringiensis* serovar pakistani T13001	4
NZ_CM000755.1	*B. thuringiensis* serovar pondicheriensis BGSC 4BA1	3
NZ_CM000757.1	*B. thuringiensis* serovar pulsiensis BGSC 4CC1	3
NZ_CM000746.1	*B. thuringiensis* serovar tochigiensis BGSC 4Y1	3
NC_022781.1	*B. toyonensis* BCT-7112	5
LOBC00000000.1	*B. wiedmannii* FSL W8-0169	2
NC_010184.1	*B. weihenstephanensis* KBAB4	6
NZ_CP009746.1	*B. weihenstephanensis* WSBC 10204	6

Supplemental Table S3. *B. cereus* group genomes used for 16S rDNA database construction.

NCBI Accession Number	Isolate
NZ_CP007666.1	*B. anthrasis* str. Vollum
NC_004722.1	*B. cereus* ATCC 14579
NC_009674.1	*B. cytotoxicus* NVH 391-98
NZ_CM000742.1	*B. mycoides* DSM 2048
NZ_CM000745.1	*B. pseudomycoides* DSM 12442
NZ_CM000753.1	*B. thuringiensis* serovar Berliner ATCC 10792
CP006863.1	*B. toyonensis* BCT-7112
LOBC00000000.1	*B. wiedmannii* FSL W8-0169
NZ_CP009746.1	*B. weihenstephanensis* str. WSBC 102014

Supplemental Table S4. *B. cereus* group isolate genomes in the training set (n = 22)

**Isolate ID**	**SRA Accession**	***rpoB* AT**	**MLST ST**	**Phylogenetic Clade**
FSL H7-0926	SRR2541537	90	667	6
FSL H8-0482	SRR2541601	129	223	5
FSL H8-0488	SRR2541602	129	111	5
FSL K6-0040	SRR2541603	362	1101	4
FSL K6-0043	SRR2541604	363	1087	4
FSL K6-0067	SRR2541605	365	1086	3
FSL K6-0069	SRR2541606	194	1080	2
FSL K6-0073	SRR2541607	366	33	4
FSL K6-0267	SRR2541613	90	1090	6
FSL M8-0117	SRR2541639	308	1085	3
FSL W8-0003	SRR2541640	125	1084	3
FSL W8-0050	SRR2541641	380	32	3
FSL W8-0169	SRR2541651	61	1081	2
FSL W8-0268	SRR2541662	92	1083	4
FSL W8-0275	SRR2541668	463	1050	3
FSL W8-0483	SRR2541674	120	1082	3
FSL W8-0520	SRR2541680	481	1032	3
FSL W8-0523	SRR2541686	481	1032	3
FSL W8-0640	SRR2541688	154	1089	4
FSL W8-0824	SRR2541693	92	24	4
FSL W8-0932	SRR2541715	120	365	3
FSL W8-0767	SRR2541718	154	787	4

Supplemental Table S5. Primers used in this study

Primer Name	Primer sequence (5' to 3')	Annealing Temperature (°C)	Reference
FHblC	CCTATCAATACTCTCGCAA	45	1
RHblC	TTTCCTTTGTTATACGCTGC	45	1
FHblD	GAAACAGGGTCTCATATTCT	45	1
RHblD2	CTGCATCTTTATGAATATCA	45	1
FHblA	GCAAAATCTATGAATGCCTA	45	1
RHblA	GCATCTGTTCGTAATGTTTT	45	1
F2NheA	TAAGGAGGGGCAAACAGAAG	52	1
RNheA	TGAATGCGAAGAGCTGCTTC	52	1
F2NheB	CAAGCTCCAGTTCATGCGG	52	1
RNheB	GATCCCATTGTGTACCATTG	52	1
FNheC	ACATCCTTTTGCAGCAGAAC	52	1
R2NheC	CCACCAGCAATGACCATATC	52	1
FCytK	CGACGTCACAAGTTGTAACA	52	1
R2CytK	CGTGTGTAAATACCCCAGTT	52	1
FEntFM	GTTCGTTCAGGTGCTGGTAC	56	1
REntFM	AGCTGGGCCTGTACGTACTT	56	1
gmkF	ATTTAAGTGAGGAAGGGTAGG	56	2
gmkR	GCAATGTTCACCAACCACAA	56	2
ptaF	GCAGAGCGTTTAGCAAAAGAA	56	2
ptaR	TGCAATGCGAGTTGCTTCTA	56	2
purF	CTGCTGCGAAAAATCACAAA	56	2
purR	CTCACGATTCGCTGCAATAA	56	2
pycAF	GCGTTAGGTGGAAACGAAAG	57	2
pycAR	CGCGTCCAAGTTTATGGAAT	57	2
ilvDF	CGGGGCAAACATTAAGAGAA	58	2
ilvDR	GGTTCTGGTCGTTTCCATTC	58	2
tpiF	GCCCAGTAGCACTTAGCGAC	58	2
tpiR	CCGAAACCGTCAAGAATGAT	58	2
glpF	GCGTTTGTGCTGGTGTAAGT	59	2
glpR	CTGCAATCGGAAGGAAGAAG	59	2

REFERENCES

1. Ngamwongsatit P, Buasri W, Pianariyanon P, Pulsrikarn C, Ohba M, Assavanig A, Panbangred W**.** 2008. Broad distribution of enterotoxin genes (*hblCDA*, *nheABC*, *cytK*, and *entFM*) among *Bacillus thuringiensis* and *Bacillus cereus* as shown by novel primers. Int J Food Microbiol 121:352-356.

2. Jolley K. 2016. *Bacillus cereus* Multi Locus Sequence Typing. http://pubmlst.org/bcereus/. Accessed 07-06-2016.

Supplemental Table S6. *B. cereus* group genomes in the validation set (n = 24)

Isolate ID	SRA Accession	*rpoB* AT	MLST ST	Phylogenetic clade
FSL H7-0344	SRR5185037	59	1097	2
FSL H7-0683		75	222	6
FSL H8-0485	SRR5189059	132	1142	6
FSL H8-0492	SRR4661790	134	1098	6
FSL H8-0534	SRR4661789	148	83	1
FSL J3-0113	SRR3458443	417	1094	2
FSL J3-0123	SRR5189058	513	1095	6
FSL K6-0042	SRR5185033	128	87	1
FSL K6-0268	SRR5185034	409	230	4
FSL M7-0109	SRR5189057	273	410	6
FSL M7-0669	SRR5189065	3	655	6
FSL M7-1219	SRR5189064	97	1092	6
FSL M8-0091	SRR5189063	410	564	2
FSL M8-0139	SRR5185026	424	138	4
FSL M8-0214	SRR5185025	412	223	5
FSL R5-0184	SRR5185020	277	927	4
FSL R5-0585	SRR5185019	278	1143	4
FSL R5-0594	SRR5185018	246	1096	3
FSL R5-0708	SRR5189062	257	1144	6
FSL R5-0859	SRR5185016	158	1099	4
FSL W7-1101	SRR5185011	296	1100	4
FSL W7-1108	SRR5189056	342	673	6
FSL W7-1328	SRR5185010	327	1093	3
FSL W7-1334	SRR5185009	304	265	4

Supplemental Table S7. Virulence genes found to be significantly associated with *B. cereus* group phylogenetic clades after a Bonferroni correction. Odds ratios less than 1 correspond to a virulence gene sharing a positive association with a clade, while odds ratios greater than 1 correspond to a negative association.

Virulence Gene	*panC* Clade	Odds Ratio	Corrected P-Value
atxA	2	Infinity; not detected in clade 2	0.027383147
atxA	3	0; detected only in clade 3	5.66E-50
atxA	4	Infinity; not detected in clade 4	1.35E-22
atxA	6	Infinity; not detected in clade 6	2.05E-05
bceT	3	62.32240062	1.05E-94
bceT	4	0.064764416	3.75E-41
bceT	5	0.059850227	1.56E-05
bceT	6	0.288422556	0.001282924
capA	2	Infinity; not detected in clade 2	0.048303228
capA	3	0.032862187	7.59E-35
capA	4	11.75717237	4.44E-13
capA	6	Infinity; not detected in clade 6	6.69E-05
capB	2	Infinity; not detected in clade 2	0.048303228
capB	3	0.027715506	2.34E-36
capB	4	14.33693639	5.16E-14
capB	6	Infinity; not detected in clade 6	6.69E-05
capC	2	Infinity; not detected in clade 2	0.049836437
capC	3	0.032001269	1.14E-35
capC	4	12.01832512	2.36E-13
capC	6	Infinity; not detected in clade 6	3.55E-05
capD	2	Infinity; not detected in clade 2	0.048064584
capD	3	0.033303227	1.95E-34
capD	4	11.62775955	8.25E-13
capD	6	Infinity; not detected in clade 6	6.45E-05
capE	2	Infinity; not detected in clade 2	0.048064584
capE	3	0.028086371	6.11E-36
capE	4	14.17992651	5.13E-14
capE	6	Infinity; not detected in clade 6	6.45E-05
cesA	4	Infinity; not detected in clade 4	0.039756874
cesB	4	Infinity; not detected in clade 4	0.039756874
cesC	6	0.140395167	7.65E-06
cesD	4	Infinity; not detected in clade 4	0.039756874

cya	2	Infinity; not detected in clade 2	0.049836437
cya	3	0; only detected in clade 3	1.34E-48
cya	4	Infinity; not detected in clade 4	5.68E-22
cya	6	Infinity; not detected in clade 6	3.55E-05
cytK2	3	3.344035241	1.61E-11
cytK2	4	0.029196618	7.28E-63
cytK2	5	10.87410105	0.001947151
cytK2	6	Infinity; not detected in clade 6	4.51E-15
hasA	2	Infinity; not detected in clade 2	0.027803859
hasA	3	0.00404891	4.12E-47
hasA	4	Infinity; not detected in clade 4	2.61E-22
hasA	6	18.54781063	0.000666843
hblA	2	0.195842768	0.01513785
hblA	3	196.0842149	6.23E-118
hblA	4	0.017511067	2.77E-49
hblA	5	0; detected in all clade 5 genomes	4.40E-06
hblA	6	0; detected in all clade 6 genomes	1.85E-15
hblB	2	0.195842768	0.01513785
hblB	3	196.0842149	6.23E-118
hblB	4	0.017511067	2.77E-49
hblB	5	0; detected in all clade 5 genomes	4.40E-06
hblB	6	0; detected in all clade 6 genomes	1.85E-15
hblC	2	0.18955875	0.013355882
hblC	3	128.5727561	1.66E-111
hblC	4	0.03188879	1.76E-44
hblC	5	0; detected in all clade 5 genomes	3.28E-06
hblC	6	0; detected in all clade 6 genomes	1.03E-15
hblD	2	0.192045241	0.013842561
hblD	3	149.0820037	7.84E-114
hblD	4	0.024545946	6.83E-47
hblD	5	0; detected in all clade 5 genomes	3.65E-06
hblD	6	0; detected in all clade 6 genomes	1.21E-15
hlyII	3	0.155082814	3.04E-27
hlyII	5	Infinity; not detected in clade 5	7.76E-07
hlyII	6	Infinity; not detected in clade 6	2.44E-17
hlyR	3	0.252140052	1.97E-15
hlyR	5	Infinity; not detected in clade 5	1.91E-08
hlyR	6	15.54490788	3.58E-13
lef	2	Infinity; not detected in clade 2	0.048890931
lef	3	0; detected only in clade 3	3.83E-48

lef	4	Infinity; not detected in clade 4	1.10E-21
lef	6	Infinity; not detected in clade 6	3.65E-05
pagA	2	Infinity; not detected in clade 2	0.048303228
pagA	3	0; detected only in clade 3	1.09E-47
pagA	4	Infinity; not detected in clade 4	2.39E-21
pagA	6	Infinity; not detected in clade 6	6.69E-05
